# Sensitive and reliable evaluation of single-cut sgRNAs to restore dystrophin by a GFP-reporter assay

**DOI:** 10.1371/journal.pone.0239468

**Published:** 2020-09-24

**Authors:** Pin Lyu, Kyung Whan Yoo, Manish Kumar Yadav, Anthony Atala, Annemieke Aartsma-Rus, Maaike van Putten, Dongsheng Duan, Baisong Lu

**Affiliations:** 1 Wake Forest Institute for Regenerative Medicine, Wake Forest University Health Sciences, Winston-Salem, North Carolina, United States of America; 2 Leiden University Medical Center, Leiden, The Netherlands; 3 Department of Molecular Microbiology and Immunology, School of Medicine, University of Missouri, Columbia, Missouri, United States of America; University of Minnesota Medical School, UNITED STATES

## Abstract

Most Duchenne muscular dystrophy (DMD) cases are caused by deletions or duplications of one or more exons that disrupt the reading frame of *DMD* mRNA. Restoring the reading frame allows the production of partially functional dystrophin proteins, and result in less severe symptoms. Antisense oligonucleotide mediated exon skipping has been approved for DMD, but this strategy needs repeated treatment. CRISPR/Cas9 can also restore dystrophin reading frame. Although recent *in vivo* studies showed the efficacy of the single-cut reframing/exon skipping strategy, methods to find the most efficient single-cut sgRNAs for a specific mutation are lacking. Here we show that the insertion/deletion (INDEL) generating efficiency and the INDEL profiles both contribute to the reading frame restoring efficiency of a single-cut sgRNA, thus assays only examining INDEL frequency are not able to find the best sgRNAs. We therefore developed a GFP-reporter assay to evaluate single-cut reframing efficiency, reporting the combined effects of both aspects. We show that the GFP-reporter assay can reliably predict the performance of sgRNAs in myoblasts. This GFP-reporter assay makes it possible to efficiently and reliably find the most efficient single-cut sgRNA for restoring dystrophin expression.

## Introduction

Duchenne muscular dystrophy (DMD) is one of the most severe muscle diseases affecting ~1 in 5000 boys and causing premature death. It is caused by mutations in the *DMD* gene encoding the protein dystrophin, which has over 3600 amino acid residues. Dystrophin links the cytoskeleton actin to the transmembrane dystroglycan complex to maintain the integrity of the membrane of the contracting muscle fibers [1]. Dystrophin deficiency causes membrane fragility and muscle degeneration. Skeletal muscle, cardiomyocytes and diaphragm muscles are all affected, patients progressively lose muscle tissue and function, and patients mostly die from respiratory failure, cardiomyopathy, and heart failure in the 2^nd^-4^th^ decade of life.

The N-terminus and the C-terminus of dystrophin are essential for its function, but parts of the central rod domain are redundant, which explains the observation that in-frame mutations that keep the N- and C-terminal domains intact are associated with a milder phenotype, observed in Becker muscular dystrophy (BMD) patients [2]. Intragenic deletions and duplications, which cause a reading frame shift and a premature translation termination, account for over two thirds of the over 8000 *DMD* mutations [3]. Restoration of the reading frame, would allow these DMD patients to produce partially functional Becker-like dystrophins, which is the principle for the exon skipping approach [4]. This technique uses antisense oligonucleotides, which target specific exons and cause them to be skipped during pre-mRNA splicing. Two exon skipping compounds have received approval from the Food and Drug Administration (USA), eteplirsen for exon 51 skipping and golodirsen for exon 53 skipping. However, due to turnover of the exon skipping compounds, reframed transcripts and dystrophin protein, repeated treatment is required. Currently, DMD patients received weekly intravenous infusions with eteplirsen and golodirsen.

Genome editing would be able to reframe mutations at the DNA level, so that repeated treatment would not be required. The CRISPR/Cas9 endonuclease can make specific DNA cuts in the human genome. After making single or double cuts, short insertion or deletions (INDELs) or long deletions may be generated after DNA repair by non-homologous end joining, the predominant DNA repair pathway. This makes CRISPR/Cas9 useful to restore dystrophin expression in cultured cells from DMD patients [5, 6]. Many groups have also explored CRIPSR/Cas9 therapy for *in vivo* DMD gene editing. This strategy has effectively restored the reading frame of the mutated dystrophin gene in mouse [7–12] and dog [13] DMD models.

**Fig 1:**
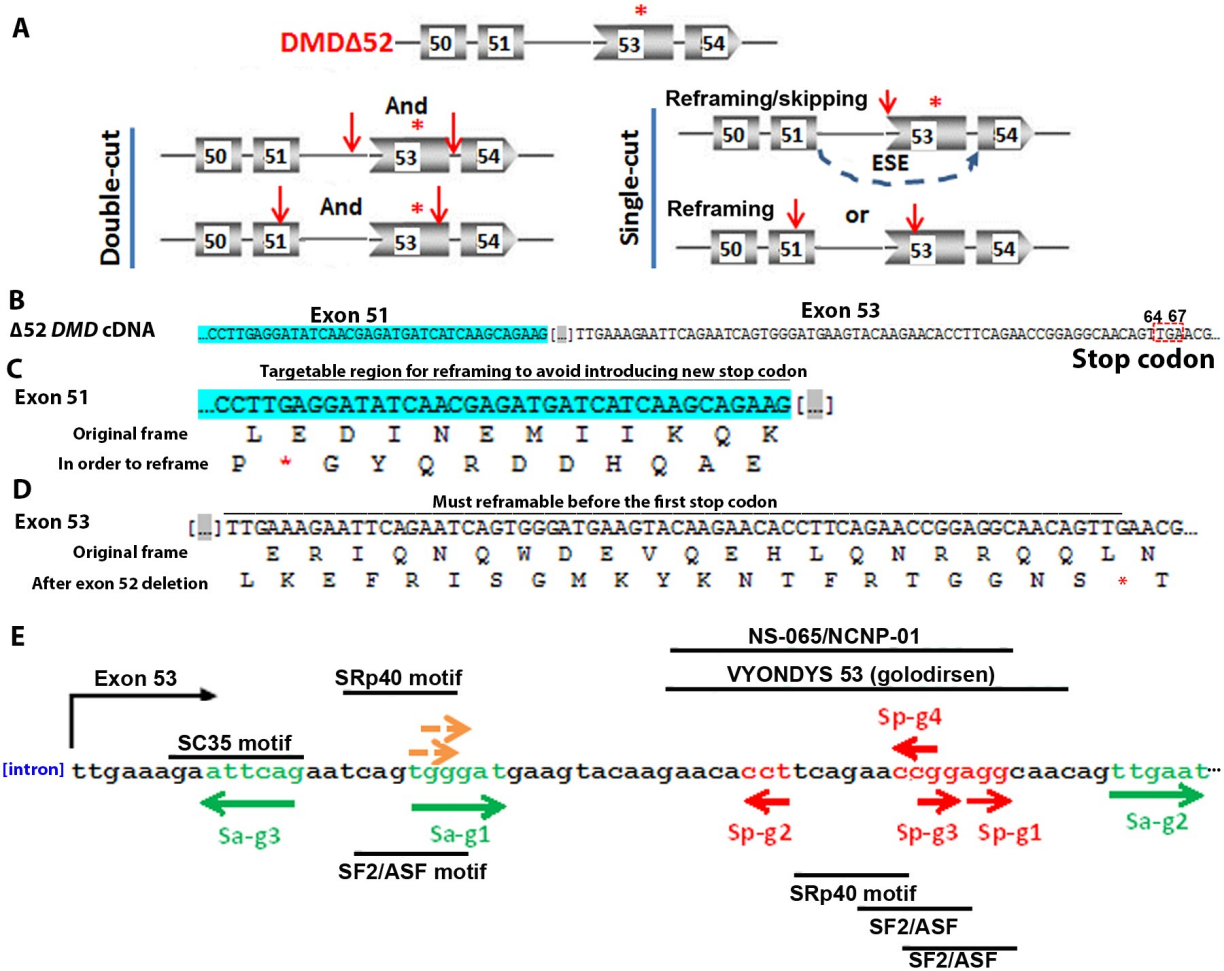
Using CRISPR/Cas9 for restoring dystrophin caused by exon 52 deletion. **A**. Single- and double-cut strategies for restoring dystrophin expression after exon 52 deletion. The asterisk in red indicates the first stop codon in the frameshift caused by exon 52 deletion. The red arrows indicate target sites of sgRNAs. Thin lines indicate introns and boxes indicate exons with numbers. “And” indicates two sgRNAs are needed and “or” indicates only one is needed. **B**. cDNA sequences of exon 51 and exon 53 near the deleted exon 52. The deleted exon 52 sequences are indicated by dots in square brackets. Exon 51 is highlighted green. The first stop codon in exon 53 caused by exon 52 deletion is indicated by a red dashed box. **C**. Sequence of exon 51 near the deleted exon 52. The original reading frame and the reading frame for reframing in exon 51 are shown. The region below the line is the targetable region that reframing does not introduce new stop codon (red asterisk) in exon 51. **D**. Sequence of exon 53 near the deleted exon 52. The original reading frame and the reading frame for reframing in exon 53 are shown. The region below the line is the targetable region to make sure that reframing happens before the first stop codon (red asterisk) in exon 53 caused by exon 52 deletion. **E**. Candidate target sites in exon 53 that can be targeted by Cas9 for reframing dystrophin for exon 52 deletion. PAM regions for SaCa9 and SpCas9 are in green and red respectively. The two brown arrows indicate the PAMs for two possible SpCas9 target sites that have too many predicted off-targets and are not pursued further. The directions of the arrows indicate whether the PAMs are on the sense strand (right directed arrows) or the anti-sense strand (left directed arrows). The target regions for VYONDYS 53 (golodirsen) and NS-065/NCNP-01 are indicated. The predicted ESE motifs near the cleavage sites are listed.

Based on the number of sgRNAs used, two strategies are applied to restore the dystrophin reading frame: the double-cut strategy and the single-cut strategy (Fig 1A). For the double-cut strategy, two sgRNAs are used that target sequences in introns flanking the exon(s) to be removed to restore the reading frame [7–9, 11, 12, 14]. For the single-cut strategy, one sgRNA is used to target exonic sequences to restore the dystrophin reading frame [10, 13, 15–17]. With this strategy, either the length of the exon is altered due to the INDELs, such that the frame is restored. Alternatively, a splice site or an exonic splicing enhancer can be disrupted, causing the skipping of this exon on RNA level, to also restore the reading frame [10, 13, 15, 16].

The single-cut strategy likely is the most promising for future clinical applications for the following reasons: 1) for the two-cut strategy two ribonucleoproteins have to be used which will increase cost; 2) for the two-cut strategy both sites have to be cleaved for the therapy to work and this requirement may reduce *in vivo* efficiency; 3) encouraging *in vivo* results have been obtained using the single-cut strategy in animal models [10, 13, 15–17]. However, not all sgRNAs are efficient in generating INDELs and a reliable screening method to find the most effective sgRNAs for dystrophin restoration would be desirable to accelerate therapy development. A single cut will generate INDELs with 3n, 3n+1 and 3n+2 base pair additions (n is any integer). For DMD however only one type of the INDELs can restore the dystrophin reading frame. In fact, the capability of a sgRNA to restore reframe dystrophin after a single cut is the combined effects of two features: INDEL generation activity and the distribution of restoring INDELs it can generate (INDEL profile). Currently the T7 endonuclease I assay and the Surveyor assay are the most commonly used methods to determine INDEL generation activities [18]. However, except for next generation sequencing, there is no simple assay to determine the INDEL distribution profiles, and which percentage is frame-restoring.

Recently several studies observed that INDEL profiles caused by a specific guide RNA are reproducibly biased and determined by the target sequence [19–21]. In other words, a sgRNA may generate unequal numbers of INDELs with 3n, 3n+1 and 3n+2 changes. Consistent with these observations, a reframing 1 nucleotide insertion was found to account for majority of restored dystrophin expression events when using single sgRNA to in cells carrying an exon 44 or a 50 deletion [10, 15]. These observations suggest that sgRNAs with similar INDEL-generation activities (assayed by T7E1 assay) may have different capacities to restore dystrophin, and a strategy to efficiently find the most effective sgRNAs for this purpose will accelerate the development of effective single-cut DMD gene editing therapies. This study was initiated to address this need.

## Results

### Designing a GFP-reporter assay for evaluating single-cut sgRNAs for dystrophin restoration

We decided to develop a GFP-reporter assay to efficiently evaluate the capacity of sgRNAs to restore dystrophin expression via reframing. For proof-of-concept, we chose to develop an assay to screen for sgRNAs restoring dystrophin expression for a *DMD* exon-52 deletion. This specific deletion was selected because a humanized mouse model, del52hDMD/*mdx*, carrying copies of human *DMD* with a deletion of exon 52 in an *mdx* background is available [22].

An exon-52 deletion causes a frame-shift and multiple stop codons, the first at 64^th^ to 67^th^ nucleotide in exon 53 (Fig 1B). For the purpose of restoring dystrophin expression by single-cut reframing, sgRNAs can be designed targeting exon 51 or exon 53. In exon 51 the sgRNAs should target a region in such a way that reframing does not introduce a new stop codon (Fig 1C), whereas in exon 53 the sgRNAs should reframe before the first stop codon introduced by the frame shift (Fig 1D). In most studies using a single sgRNA to restore dystrophin, the sgRNAs were designed to target the exonic splicing enhancer (ESE) to induce exon skipping in addition to INDEL-mediated reframing [10, 13, 15, 16]. Since reframing events were observed three times more frequently than exon skipping in these studies even when the ESEs were targeted, we searched for all possible single sgRNA target sites for SaCas9 and SpCas9 within the target exons. Considering the demonstrated low cleavage efficiency of targets with non-canonical protospacer adjacent motifs (PAM), such as NAG for SpCas9 [23], we only considered the target sites with canonical PAM sequences (NGG for SpCas9 and NNGRRT for SaCas9, R indicates A or G).

A sequence analysis with CRISPOR [24] found two SpCas9 targets in exon 51, and 5 SpCas9 targets and 3 SaCas9 targets in exon 53 that can be used to reframe dystrophin for an exon 52 deletion (Fig 1E). Due to the predicted high amounts of off-targets, two of the SpCas9 targets in exon 53 (Fig 1E, PAMs indicated by brown arrows) were not pursued. Considering that there are only two candidate sgRNAs in exon 51, and that targeting exon 53 can also treat patients with exons 45–52, 47–52, 48–52, 49–52, 50–52 deletions, we decided to focus on sgRNAs in exon 53 for proof of concept. This left us a total of 7 sgRNAs for evaluation, three for SaCas9 and four for SpCas9. ESEfinder software [25] and Human Splicing Finder (http://www.umd.be/HSF/) indicated that the cleavage sites of all sgRNAs except Sa-gRNA2 fall into at least one predicted ESE motifs, and even that of Sa-gRNA2 was only two bps away. Except for Sa-gRNA1 and Sa-gRNA3, the target sequences of all other sgRNAs overlapped with the target regions of a recently approved antisense drug VYONDYS 53 (golodirsen, https://www.rxlist.com/vyondys-53-drug.htm#description) and an antisense oligo NS-065/NCNP-01 under development [26].

**Fig 2:**
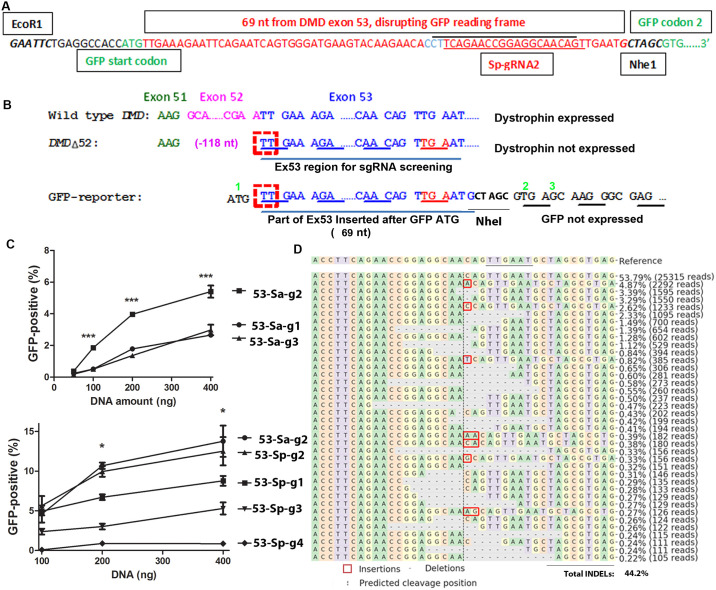
A GFP-reporter assay for evaluating sgRNAs for *DMD* reframing by single sgRNA cut after exon 52 deletion. **A**. Design of the GFP-reporter cassette. The first and the second codons of GFP are shown green. The inserted sequences from *DMD* exon 53 are shown in red. The target sequences for Sa-gRNA2 (below the black line) and for Sp-gRNA2 (above the red line, the PAM region is shown in blue) are indicated. The two restriction enzyme sites added for sub-cloning are also shown. **B**. Comparison of the reading frames of the disrupted GFP and DMD. The normal dystrophin reading frame (top) and the reading frame shift caused by exon 52 (pink) deletion are shown. The insertion of 74 nt (69 nt from exon 53 and the rest 5 nt to make a NheI enzyme site for possible other target cloning) after the GFP start codon creates the same frame shift to GFP as the deletion of exon 52 to dystrophin. A deletion of 3n+2 nucleotides (exampled by the red dashed boxes when n = 0) will restore reading frame for both. Green numbers indicate amino acid numbers for the wild type GFP. **C**. Comparing SaCas9 (top) and SpCas9 gRNAs (bottom) by transfecting GFP-reporter cells. *** indicates p<0.0001 when Sa-gRNA2 was compared with other Sa-gRNAa (ANOVA). * indicates p<0.05 when Sa-gRNA2 was compared with SpCas9 gRNAs other than Sp-gRNA2 (ANOVA). Increasing amount of DNA was transfected into 1.25x10^5^ GFP-reporter cells and the cells were analyzed by flow cytometry 48 hours after transfection. Three replicates were included for each condition. Shown were representative data of at least two independent experiments. **D**. Next generation sequencing (NGS) analysis of INDELs in the target region of GFP-reporter cassette. The GFP-reporter cells (1.25x10^5^) were transfected with 0.5 μg plasmid DNA co-expressing SaCas9 and sgRNA Sa-gRNA2. The target DNA in the GFP-reporter cassette was amplified with primers (reporter-F1 and reporter-R2) for sequencing analysis. The original sequence (reference) is listed on the top line with the protospacer adjacent motif (PAM, NNGRRT for SaCas9, “N” can be any nucleotide, and “R” is A or G) underlined. Below the reference sequence are listed types of readings over 0.2% observed in NGS. Reading number and percentage of each type of reading are listed at the right of that sequence. Note that all INDELs are around the predicted cleavage site (vertical dashed line).

In order to quantitatively compare the activities of sgRNAs for dystrophin reframing, we designed a GFP-reporter assay. We inserted 74 nt after the GFP start codon, 69 nt of which are derived from *DMD* exon 53 (from the first 5’ nucleotides of *DMD* exon 53 to the stop codon caused by an exon 52 deletion) (Fig 2A). The GFP-reporter cassette was flanked by EcoRI and NheI sites to facilitate making reporter cassette for other targets. The sequence inserted between GFP codon 1 and 2 contains all possible sgRNA target sequences listed in Fig 1E. One of the sgRNAs, Sp-gRNA2, was marked as an example. This insertion creates a frame shift for GFP with a 3n+2 insertion. This frame shift matches the frame shift in the hDMDdel52/mdx mouse (Fig 2B). Without INDELs, GFP will not be expressed due to the frame shift. The sgRNAs that are highly active and preferably generate the types of INDELs that can restore GFP-expression (deletions of 3n+2) will generate a large number of GFP-positive reporter cells, which can be easily assayed by GFP-based flow cytometry. Since the GFP-expression cassette and the hDMDdel52 transgene have the same types of reading frame shifts, we reasoned that INDELs most efficiently restore GFP expression from the GFP-expression cassette will also most efficiently restore dystrophin expression from in human cells and the hDMDdel52/*mdx* mouse.

We made lentiviral vectors containing the GFP-reporter cassette, transduced HEK293T cells with the lentiviral vectors and selected cells with integrated reporter cassette by adding puromycin to the culture medium (the cDNA coding for puromycin resistant N-acetyl-transferase is linked to the GFP cDNA by internal ribosome entry site sequence). Untreated cells and cells transfected with DNA co-expressing SaCas9 and *IL2RG* sgRNA1 [27] (an unrelated sgRNA targeting *IL2RG*) produced no GFP-positive cells (S1 Fig), consistent with GFP reading frame disruption by the target DNA insertion. When the cells were transfected with plasmid DNA co-expressing SaCas9 and Sa-53-gRNA2 (targeting sequence below the black line in Fig 2A), one of the sgRNAs targeting *DMD* exon 53 and the inserted sequence in the GFP-reporter cassette, we observed GFP-positive cells (S1 Fig). Flow cytometry analysis showed a dose-dependent increase of GFP-positive cells (Fig 2C, S2 Fig). These observations suggest that our GFP-reporter cells were functional and GFP expression was most likely the result of INDEL-caused reframing. Next generation sequencing revealed INDELs in the target sequence of the GFP reporter cassette (Fig 2D).

We made constructs co-expressing Cas9 and various *DMD* exon 53-targeting sgRNAs (three SaCas9 sgRNAs and four SpCas9 sgRNAs), and transfected the plasmid DNA into the GFP-reporter cells. The percentages of GFP-positive reporter cells were compared by flow cytometry. We found that different sgRNAs showed quite different activities in generating GFP-positive reporter cells. Sa-gRNA2, Sp-gRNA1 and Sp-gRNA2 consistently generated more GFP-positive cells than the other sgRNAs in transfection experiments (Fig 2C, S2 Fig). The data showed that the GFP-reporter cells can efficiently differentiate sgRNAs in their capability to generate GFP-positive cells.

#### Single guide RNAs showed reproducible and sgRNA-specific INDEL profiles

We amplified the target DNA region of the GFP-reporter cassette from cells treated by the three best performing gRNAs (Sa-gRNA2, Sp-gRNA1 and Sp-gRNA2) and analyzed it by NGS. Cells with similar percentages of GFP-positive cells (~20%, transfected with different amounts of DNA) were analyzed. NGS analysis found that although the total INDEL rates were 44.2%, 54% and 37.8% for Sa-gRNA2, Sp-gRNA1 and Sp-gRNA2 respectively, the restoring INDELs (those with 3n+2 deletions or 3n+1 insertions that can restore GFP reading frame) consisted of 20% of the total for each gRNA (Table 1), explaining the observation of about 20% GFP-positive cells. This observation also showed the causative role of INDEL generation in GFP-expression. It is interesting that a high total INDEL rate did not result in a high percentage of GFP-positive cells, confirming that INDEL activity is not a good indicator for a sgRNA’s reading frame restoring activity.

**Table 1:** INDEL profiles of sgRNAs when transfecting plasmid DNA or transducing RNPs.

Guide RNA	Plasmid transfection (n = 1)			RNP (n = 3)		
	Restoring (Actual %)	Non-restoring (Actual %)	Restoring (Relative %)^b^	Restoring (Actual %)	Non-restoring (Actual %)	Restoring (Relative %) ^b^
Control^a^	0.08	0.16	ND			
Sa-gRNA2	19.5	24.7	44.1	20.5±4.9	32.9±6.5	37.8±1.1
Sp-gRNA1	21	33	38.9	19.1±1.1	34.5±3.4	35.9±3.4
Sp-gRNA2	21.4	16.4	56.6	31.8±2.5	21.7±1.7	59.9±0.8***

^a^Control DNA were from cells transfected with plasmid expressing SaCas9 and sgRNA targeting *IL2RG*.

^b^Relative percentage is the percentage of restoring INDELs out of all INDELs observed in that sample. Mean±SEM are listed.

*** indicates p<0.0001 compared with Sa-gRNA2 or Sp-gRNA1 (Tukey’s Multiple Comparison Test following ANOVA).

It was observed that CRISPR/Cas9 delivered by DNA transfection could have different behaviors than those delivered as ribonucleoproteins (RNPs) [28]. Considering that future clinical CRISPR editing of DMD will likely use RNPs, we used our lentiviral capsid-based Cas9 RNP systems [29] (and Lu et al, unpublished) to further compare the four best performing sgRNAs (Sa-gRNA1, Sa-gRNA2, Sp-gRNA1 and Sp-gRNA2). The target site of Sa-gRNA1 was closer to the intron/exon junction.

Lentivirus-like particles (LVLPs) containing Sa-gRNA1, Sa-gRNA2, Sp-gRNA1 and Sp-gRNA2 RNPs were prepared and used to transduce our GFP-reporter cells (100 ng p24 LVLPs for 2.5x10^4^ cells). When delivered as RNPs, they generated 3.9%±0.2%, 19.5%±0.1%, 18.5%±0.6% and 30.3%±0.3% GFP-positive cells respectively. Except for Sa-gRNA2 and Sp-gRNA1 RNPs, which showed no significant difference, comparisons between other RNPs all showed a significant difference (Tukey’s Multiple Comparison Test after ANOVA. *P*<0.0001, F = 963.4, R squared 0.9952). Note that Sa-gRNA1 RNPs only generated 1/5 GFP-positive cells compared with Sa-gRNA2 RNPs, whereas in DNA transfection experiments Sa-gRNA1/Cas9 plasmid DNA generated half numbers of GFP-positive cells generated by Sa-gRNA2/Cas9 DNA. The most likely explanation is that unlike in DNA transfection where Cas9/sgRNA expression lasts for several days, RNPs have short half-life and thus will better reflect the activity difference of different sgRNAs. This observation also shows the benefit of using RNPs to evaluate sgRNA performance.

Since Sa-gRNA2, Sp-gRNA1 and Sp-gRNA2 RNPs generated high percentages of GFP-positive cells, we further analyzed the INDELs generated by these three RNPs by NGS (Table 1). The overall INDEL rates for Sa-gRNA2, Sp-gRNA1 and Sp-gRNA2 RNPs were 65.2±0.4%, 53.3±2.8% and 53.2±4.5% respectively. Although Sa-gRNA2 generated the highest level of overall INDELs (65%), Sp-gRNA2 RNPs generated more frame-restoring INDELs (31.8%) and thus more GFP-positive cells (Table 1, S3–S5 Figs). Sp-gRNA2 constantly had high percentages of restoring INDELs out of total INDELs (59.9%), over 20% higher than those of the other two sgRNAs. Sequence analyses of the DNA from cells treated with different sgRNAs confirmed that the reading frame restoring activity of a sgRNA was the combined effects of total INDEL rate and the percentage of frame-restoring INDELs. The GFP-reporter assay but not the T7E1 assay is suitable for detecting the reading frame restoring activity.

#### The GFP-reporter assay predicted the performance of sgRNAs in myoblasts

The GFP-reporter assay will be useful only if it can predict the performance of sgRNAs in myoblasts. We thus examined the INDELs of the sgRNAs in myoblasts. Although the LVLP-RNPs could have a different transduction efficiency and INDEL rate between HEK293T cells and primary myoblasts, the focus of this experiment was to assess whether the INDEL profiles of the same sgRNA remain consistent in the two cell types.

**Fig 3:**
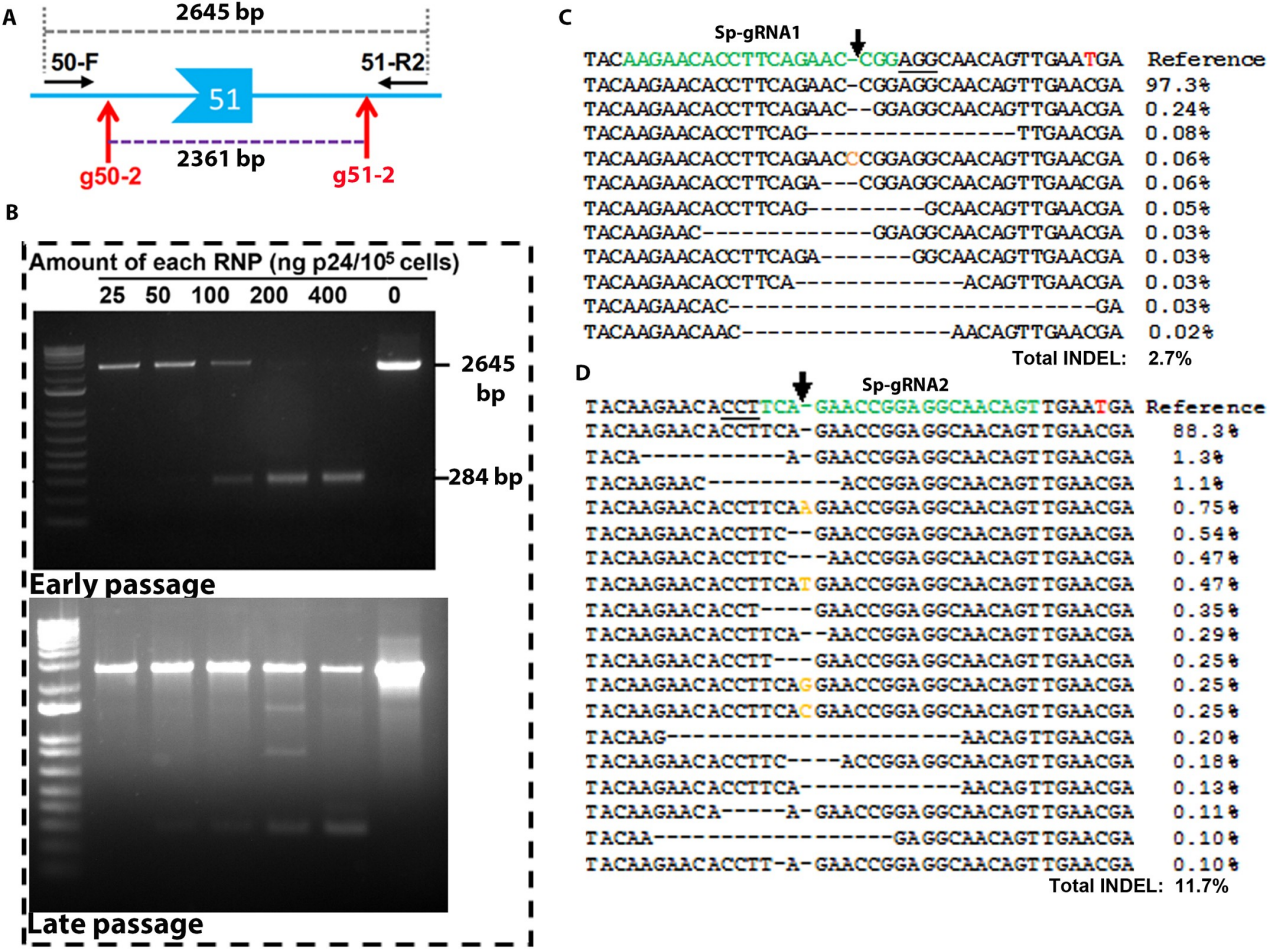
LVLP-RNPs were functional in myoblasts. **A**. Cartoon illustrating the location of the gRNAs (g50-2 and g51-2) used for exon 51 removal and the primers (DMD50-F and DMD51-R2) used for PCR detection of exon 51 removal. **B**. Test the transduction of myoblasts by LVLP-RNPs. Top: efficient removal of exon 51 in early passage hDMDdel52/*mdx* mouse myoblasts. Indicated amounts of LVLP-RNPs were co-delivered to 10^5^ myoblasts prepared from hDMDdel52/*mdx* mice. PCR yielded a 2645 bp band if exon 51 was not removed. PCR yielded a 284 bp band if exon 51 was removed. Nearly 100% efficiency of exon 51 removal could be obtained at the doses of 200 ng and 400 ng p24. Bottom: less efficient removal of exon 51 in hDMDdel52/*mdx* myoblasts by the LVLP-RNPs after the myoblasts had been cultured for one week. See S6 Fig for sequencing data. **C**. NGS analysis of hDMDdel52/*mdx* myoblasts gDNA treated with Sp-gRNA1 targeting LVLP-RNPs. **D**. NGS analysis of hDMDdel52/*mdx* myoblasts gDNA treated with Sp-gRNA2 targeting LVLP-RNPs. For **C** and **D**, the target sequences are in green and the PAM regions are underlined. The brown letters are insertions and the dashes are deletions. The red “T” in the reference sequence is a “C” in hDMDdel52/*mdx* myoblasts that disrupts the target site for Sa-gRNA2.

We first tested whether the LVLP-RNPs could transduce primary myoblasts. We isolated myoblasts from hDMDdel52/*mdx* mice that have copies of the human *DMD* gene with exon 52 deleted integrated in mouse chromosome 5, and treated the cells with two LVLP-RNPs targeting *DMD* intron 50 and 51. A pair of PCR primers (DMD50-F and DMD51-R2) outside of the two target sites will generate a PCR fragment of 2645 bp. However, if both RNPs worked to remove a fragment between the two targets sites, an amplicon of 284 bp could be generated (Fig 3A). Our PCR showed that these LVLP-RNPs were able to transduce myoblasts and efficiently remove exon 51, generating a smaller PCR product of 284 bp (Fig 3B). Sequencing analysis confirmed that this band was the products of gene deletion (S6 Fig for sequencing data and the sequence of the 284bp band). We then transduced the myoblasts with Sa-gRNA2, Sp-gRNA1 and Sp-gRNA2 RNPs. After myoblasts were differentiated, we extracted the DNA from the cells and amplified the human *DMD* exon 53 sequences for NGS. The cells treated with RNPs targeting intron 50 and 51 were used as negative controls. We observed an INDEL rate of 0.10% in these negative control cells. Sa-gRNA2 RNPs generated similar background levels of INDELs (0.11%), due to a sequence polymorphism in hDMDdel52/*mdx* mice disrupting the PAM for Sa-gRNA2 [TTGAAT in human reference genome, but TTGAAC in hDMDdel52/*mdx* mice, disrupting the canonical SaCas9 PAM of NNGRRT (R is A or G)]. Sa-gRNA2 RNPs were thus not pursued further.

We focused on INDELs generated by Sp-gRNA1 and Sp-gRNA2 RNPs. We observed variant but dose-dependent overall INDEL rates (Table 2). These INDELs were the specific products of the RNPs, since they were observed around the predicted cleavage site (Fig 3C and 3D). Chi-square analyses also observed significant differences in the observed INDEL events between control and specific RNP-treated samples. In this experiment, the overall INDEL rates were not as high as observed in the GFP-reporter cells. One reason could be that different cells have different transduction efficiency by the RNP particles. Another reason was that the myoblasts had been cultured for one week for practical reasons before treated by the particles, and we found that prolonged culture reduces the transduction efficiency by the particles (Fig 3B). However, these INDEL rates still allowed us to compare INDEL profiles.

**Table 2:** Single guide RNA profiles in myoblasts^a^.

	*DMD 50 sgRNAs*	*DMD* 53 Sa-gRNA2	*DMD* 53 Sp-gRNA1			*DMD* 53 Sp-gRNA2		
LVLP-RNPs (ng p24)	400	400	100	200	200	100	200	400
Total Readings	80718	95391	39032	44434	41688	36037	41562	88076
Readings with INDELs	95	109	512	998	1137	870	4987	10294
P (Chi square)	N/A	0.89	<0.0001	<0.0001	<0.0001	<0.0001	<0.0001	<0.0001
Total INDEL %	0.12%	0.11%	1.3%	2.24%	2.7%	2.4%	4.8%	11.7%
Restoring (Relative %)^b^	N/A	N/A	28.5%	37.6%	26.7%	53.8%	52.0%	49.9%

^a^Indicated amounts of lentiviral capsid packaged Cas9 ribonucleoprotein (ng p24) were delivered to 1x10^5^ myoblasts isolated from hDMDdel52/*mdx* mice. The target sequences of human DMD exon 53 amplified from gDNA were analyzed by NGS.

^b^Significantly different between Sp-gRNA1 and Sp-gRNA2 (t-test, p<0.05).

We calculated the rates of dystrophin restoring INDELs versus non-restoring INDELs (Table 2), and found that in myoblasts, over 51.9±1.1% of all INDELs from Sp-gRNA2 RNPs were dystrophin-restoring INDELs, and while only about 30.9±3.4% of all INDELs from Sp-gRNA1 RNPs were dystrophin-restoring INDELs. These numbers were similar to those observed in GFP-reporter cells. In both cell types, Sp-gRNA2 produced a higher percentage of restoring INDELs than Sp-gRNA1 did. The data showed that sgRNAs had similar INDEL profiles in GFP-reporter cells and myoblasts, and the performance in GFP-reporter cells was consistent with the performance in myoblasts.

#### *DMD* cDNA analyses further validated the GFP-reporter assay

**Fig 4:**
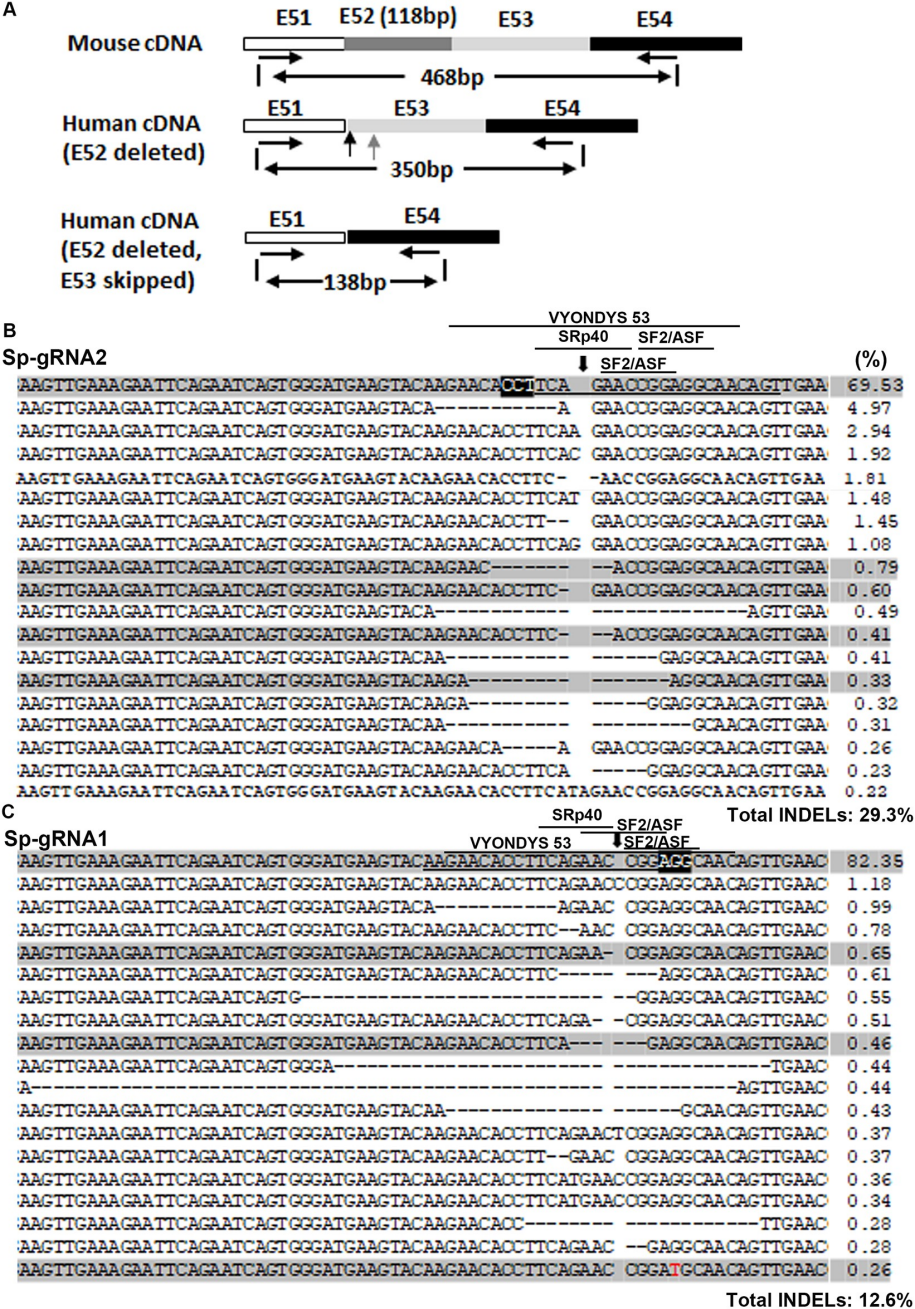
NGS analysis of cDNA from hDMDdel52/*mdx* myoblasts treated with various RNPs. **A**. Diagram showing the strategy of PCR amplification of human *DMD* cDNA for NGS. The forward and reverse PCR primers were DMD51-EX-F and DMD54-R1. PCR products of 100–350 bp were recovered for NGS, so that the mouse *Dmd* derived PCR products (468 bp) were not analyzed. **B**. Analysis of cDNA sequences without exon 53 skipping after Sp-gRNA1 RNP treatment. **C**. Analysis of cDNA sequences without exon 53 skipping after Sp-gRNA2 RNP treatment. For (**B** and **C**), myoblasts (1x10^5^) were treated with each 120 ng p24 of LVLP-RNPs. The human *DMD* target sequence was amplified by strategy shown in **A** for NGS. The PAM regions are highlighted black and the target sequences are underlined. The predicted cleavage sites are indicated by a vertical arrow. Dashed lines indicate deletions. Substitutions are in red. Numbers at the right are the percentages of the alleles. The shaded sequences are those did not reframe dystrophin. The predicted high score ESE motifs and the target region for VYONDYS 53 (golodirsen) were indicated by lines.

Our GFP-reporter assay was unable to detect the potential exon skipping activity caused by targeting the ESE. Since the cleavage sites of these sgRNAs were either within predicted ESE motifs or 2 bp away from ESE motifs, we wondered whether their capacities of exon skipping correlate with their INDEL-generation activities. We compared exon 53 skipping caused by Sa-gRNA1, Sp-gRNA1 and Sp-gRNA2. The LVLP-RNPs containing the three sgRNAs were used to transduce myoblasts from hDMDdel52/*mdx* mice. mRNA was isolated from the treated myoblasts, followed by RT-PCR and NGS analysis of the cDNA. In order to detect possible cDNAs with exon 53 skipping, the forward and reverse primers were designed to match exon 51 and exon 54 respectively (Fig 4A). Due to the sequence similarity between mouse *Dmd* and human *DMD* cDNA, mouse *Dmd* cDNA could also be amplified. However, human *DMD* cDNA amplicons were 118 bp smaller than the mouse amplicons due to the exon 52 deletion (350 bp for human versus 468 bp). If exon 53 is skipped in human *DMD* cDNA, then the PCR products will be 138 bp. We thus recovered the PCR products of sizes from 100 to 350 bp from the gel, so that cDNA amplicons with exon 53 skipping could be recovered.

We then compared exon 53 skipping in cells treated with RNPs containing the three gRNAs by NGS analysis of cDNA (Table 3). In order to get an idea of background levels of mRNAs with exon 53 skipping in myoblasts from hDMDdel52/*mdx* mice, we analyzed cDNA from cells treated with RNPs targeting human *BCL11A* enhancer sequence (sgRNA-1617) [30], whose target was unrelated to *DMD* exon 53. We observed an unexpected 54.3% cDNA molecules with exon 53 skipping. Similar analysis of cDNA from early passage myoblasts isolated from the same mouse observed 31.9% cDNA molecules with exon 53 skipping. It has been reported that spontaneous exon skipping to restore the reading frame can occur in DMD patients [31], though not at these levels. Considering that these myoblasts were isolated from a single mouse, we analyzed cDNAs produced from muscle tissues of three more mice to see whether background exon 53 skipping could be observed in other mice. We observed an average of 21.7% molecules with exon 53 skipping. The data showed that this cohort of hDMDdel52/*mdx* mice spontaneously generated various levels of cDNAs with exon 53 skipping and restored dystrophin reading frame, consistent with recent report that hDMDdel52/*mdx* mice had background dystrophin expression [14]. At present we are unclear whether all cells express mRNA with exon 53 skipping or only part of the cells do, and whether cDNA with exon 53 skipping arise from DNA mutation or alternative splicing. Since we did observe cDNAs without exon 53 skipping in all animals tested, exon skipping caused by targeting ESE should be observed. However, we observed 58.5%, 56.4% and 45.3% cDNAs with exon 53 skipping in cells treated with RNPs containing Sa-gRNA1, Sp-gRNA1 and Sp-gRNA2 (Table 3). Due to the observation of 54.3% cDNA with exon 53 skipping in cells treated with RNPs targeting an unrelated sequence, we did not detect an obvious increase of exon 53 skipping in cells treated with any of the exon 53-targeting gRNAs, even though Sp-gRNA1 and Sp-gRNA2 targeted the same target region for golodirsen and NS-065/NCNP-01 [26]. We observed even lower percentages of cDNA with exon 53 skipping from Sp-gRNA2 RNP treated cells, this could be caused by more cDNAs without exon 53 skipping whose reading frame was restored by INDELs in exon 53 (see below).

**Table 3:** cDNA with exon 53 skipping and INDEL rates in cDNA with exon 53.

	% of cDNA with exon 53 skipping	cDNA without exon 53 skipping	
		Total INDELs (%)	Restoring INDELs (%)^a^
Muscle tissue	21.7±6.4 (n = 3)	0.37±0.12 (n = 3)	ND
Non-targeting RNP	54.3 (n = 1)	1.4 (n = 1)	1.4 (n = 1)^b^
Sa-gRNA2 RNPs	58.5±3.6 (n = 3)	5.3±0.95 (n = 3)	3.5±0.45 (n = 3)
Sp-gRNA1 RNPs	56.4± 2.9 (n = 2)	11.2±1.5 (n = 3)	6.1±1.0 (n = 3)
Sp-gRNA2 RNPs	45.3±4.8 (n = 2)	25.9±2.2 (n = 3)**	16.1±2.2 (n = 3)**

^a^Calculated based on the top 20 most frequently observed readings, accounting for 85%~95% of all readings.

^b^The two types of INDELs accounted for all observed INDELs, all restored dystrophin reading frame.

** indicates p<0.01 when compared with values of Sp-gRNA1 or Sa-gRNA1 (Tukey’s Multiple Comparison Test following ANOVA).

We then focused on the cDNAs without exon 53 skipping, which enabled us to examine the INDELs generated by various RNPs. In cells treated with non-relevant RNPs, we observed 1.4% cDNA readings with INDELs. These INDELs included only two types of sequences: one with 1 bp insertion and one with 11bp deletion, both restored dystrophin. These INDELs could be caused by clonal expansion after spontaneous mutation restoring dystrophin reading frame, and these mRNAs were enriched in cDNA because they were protected from nonsense-mediated mRNA decay. Similar to observed GFP-reporter cells and myoblast DNA analysis, we observed more overall INDELs in cells treated with Sp-gRNA2 RNPs, followed by cells treated with Sp-gRNA1 RNPs and cells treated with Sa-gRNA1 RNPs (Table 3). The INDELs generated by Sp-gRNA1 (Fig 4B) and Sp-gRNA2 (Fig 4C) were all around the predicted cleavage sites (Fig 4B), supporting that they were generated from the specific endonucleases. Thus, cDNA analyses also confirmed our observations made in the GFP-reporter assays that Sp-gRNA2 was the best single-cut sgRNA for restoring GFP/dystrophin after exon 52 deletion, and that Sa-gRNA1 was inefficient in INDEL generation thus the possible additional benefits of causing exon skipping were negligible.

We then examined whether cDNA sequences with damaged ESE motifs could be observed. We observed many readings with at least one high score ESE motif damaged, and some readings had the whole VYONDYS 53 (golodirsen) target region deleted (Fig 4B and 4C). If damaging these ESE at the DNA level impaired splicing of exon 53, we should not observe these readings in cDNA due to the skipping of exon 53. The observation of these cDNA sequences suggested that at least for exon 53, CRISPR/Cas9 is not efficient in causing exon skipping. The data showed that CRIPSR/Cas9 was more efficient in producing INDELs to reframe dystrophin than targeting ESE for exon skipping. In other words, only considering sgRNAs targeting exonic splicing enhancer sequences has the risks of missing more efficient sgRNAs for restoring dystrophin.

## Discussion

Currently, DMD gene editing strategies include using two sgRNAs for partial [14] or whole exon removal [7–9, 11, 12], and using a single sgRNA for exon skipping and/or reframing [10, 13, 15–17]. In clinical applications, the double-cut strategies is associated with increased production cost since RNPs targeting two different target sites need to be prepared, and decreased efficiency since only successfully cutting both sites will the therapy work. In addition, current evidence suggest that Cas9 RNPs act in a single turnover manner [32–35], simultaneously targeting two sites requires delivering more RNPs per cell, increasing risks of inducing immune responses. Furthermore, having two sgRNAs and more RNPs also increases the chances of off-targets.

The single-cut strategy does not have the challenges described above; importantly studies in rodents and canines demonstrated its efficacy [10, 13, 15–17]. However, currently there are no methods other than NGS to evaluate sgRNA reframing activity. The surveyor assay or the T7E1 assay can only measure the INDEL generating activity, which is a poor indicator of a sgRNA’s reframing activity. NGS is able to assay INDEL generating activity and reframing capability but is expensive and time consuming. This study was designed to address this unmet need.

We developed a GFP-reporter assay to reliably and efficiently screen for sgRNAs for reframing dystrophin after mutations causing stop codons, in this study we used exon 52 deletion as a proof of concept. We inserted the *DMD* target sequence to be studied after GFP start codon to create the same frame shift to GFP as exon 52 deletion to dystrophin, and screened for *DMD* exon 53 targeting sgRNAs most efficiently reframe GFP in the GFP-reporter cells. We made the following observations: 1) the capability of a sgRNA to reframe GFP or dystrophin was the combined effects of two aspects: the INDEL generating activity and the profile of the INDELs (the percentage of INDELs that can restore the reading frame). We observed that Sa-gRNA2 RNPs generated higher overall INDEL levels than Sp-gRNA2 RNPs, however they constantly generated less GFP-positive cells than Sp-gRNA2 RNPs did. Thus, using surveyor assay or T7E1 assay to analyze INDEL efficiency is not able to find the best sgRNAs for single-cut reframing.

2) The INDEL generating activity and the INDEL profiles could be greatly different between different sgRNAs, thus screening for all possible candidate sgRNAs is necessary to find the most efficient sgRNA for reframing.

3) At least in targeting exon 53 for reframing dystrophin, damaging ESE did not cause efficient exon skipping. Yet INDEL-mediated reframing was able to generate 16% dystrophin reframed cDNA at the conditions used. This observation was also consistent with previous reports that reframing contributed much more than exon skipping even the sgRNAs were designed to target the ESE [10, 13, 15, 16]. Thus only examining predicted ESE targeting sgRNAs may miss the most efficient candidates for dystrophin restoration.

4) The GFP-reporter assay described in this study can efficiently and reliably evaluate single-cut sgRNAs for restoring dystrophin expression. We showed that the INDEL profiles of a sgRNA were very similar in GFP-reporter cells and myoblasts. Although the same sgRNA may have different INDEL generating activity in the two cell types, the relative INDEL generation activities of different sgRNAs were conserved in the two cell types: sgRNAs active in GFP-reporter cells were also active in myoblasts. Importantly, the INDEL profiles of the same sgRNA were conserved in both cell types. These observations ensured the usefulness of the GFP-reporter assay. With this efficient screening assay, as many as possible candidate sgRNAs (for SaCas9 and SpCas9) can be evaluated and finding the best sgRNA for most efficient single sgRNA reframing can be achieved with reasonable resource and efforts.

One of the limitations of this assay is that it cannot detect exon skipping. Targeting ESE might cause exon skipping, thus both exon skipping and INDEL-mediated reframing could restore dystrophin reading frame, as demonstrated in recent studies [10, 13, 15, 16]. Our GFP-reporter assay cannot detect the possible exon skipping activity besides INDEL-mediated reframing. However, we showed that although the predicted cleavage sites of the sgRNAs tested fell into or were close to predicted ESE, we failed to detect evident exon skipping activity. Although the background levels of mRNAs without exon 53 in hDMDdel52/*mdx* myoblasts made it hard to detect possible low activities of exon 53 skipping from ESE targeting, we detected 16% of INDEL-reframed cDNA from the most efficient sgRNA (Sp-gRNA2). Note that this sgRNA targeted the same target region for golodirsen and NS-065/NCNP-01 [26], its predicted cleavage site hits an ESE motif with high score, and we observed cDNA sequences with the whole VYONDYS 53 target region deleted. The data suggested that for restoring dystrophin, more attention could be paid to CRISPR/cas9’s reframing activity rather than the exon skipping activity. Our data also showed the necessity of including candidate sgRNAs that do not target predicted ESEs for screening.

It has been shown that nucleosome and heterochromatin inhibit Cas9 function [36–38]. In our GFP-reporter cells the target sequences are integrated into the genome via lentiviral vectors. Since lentiviral integration targets are usually highly accessible by Cas9 [39], our assay did not consider chromatin accessibility of the target sites. However, the target sites of the sgRNAs in comparison are from the same gene and most likely from the same exon, they will have very similar chromatin accessibility. Thus the activities of sgRNAs will mostly be determined by their sequence. This explains why the most efficient sgRNA found in the GFP-reporter assays are also the best one in myoblasts.

We are yet to demonstrate dystrophin restoration at the protein level. However, technical issues prevented us from doing so. We observed that the hDMDdel52/*mdx* mice show spontaneous exon 53 skipping, which generates about 50% mRNA missing both exon 52 and 53. The reading frame of these Δ52–53 mRNAs is restored and causes the expression of a dystrophin protein that is almost indistinguishable from the dystrophin proteins restored from reframing by the sgRNAs. The background dystrophin protein (S7 Fig) makes it difficult for us to detect possible additional dystrophin protein from gene editing. However, the observation of reframing at the genomic DNA and mRNA level should enable us to conclude that the method can distinguish sgRNAs for DMD restoration. A mouse model with no spontaneous exon 53 skipping is needed for us to show this at the protein level.

Our data in no means indicate that ESE-targeting is not an important issue to be considered when designing sgRNAs for restoring dystrophin expression. When ESE-targeting sgRNAs have comparable activity with non-ESE-targeting sgRNAs in GFP-reporter assays, further evaluation in myoblasts would be necessary to find the most efficient sgRNA. Due to the simplicity and the reliability of the GFP-reporter assay, all candidate sgRNAs can be reliably and efficiently evaluated by the GFP-reporter assay, no matter whether they target ESE. Once the GFP-reporter assay narrows down all candidate sgRNAs to a best performing ESE-targeting sgRNA and a best performing non-ESE-targeting sgRNA, the two sgRNAs can be further evaluated in myoblasts by NGS. On the other hand, if the ESE-targeting sgRNAs are less than 50% efficient in GFP-reporter assays compared with the non-ESE-targeting sgRNAs, they are most likely less efficient in myoblasts since available data showed that exon skipping only contributed to less than 25% of the overall restoring events for ESE targeting sgRNAs [10, 13, 15, 16]. With the GFP-reporter assay described in this work, we will have an increased chance to find the most efficient single-cut sgRNA that can restore dystrophin expression, which may or may not target the ESE. Expanding the targeting sites beyond the intron/exon junction site will greatly increase the number of candidate sgRNAs and the chance of finding highly efficient ones.

In summary, now that the single sgRNA strategy is promising for DMD gene editing based on recent encouraging *in vivo* data using single sgRNA [10, 13, 15–17], the GFP-reporter assay described in this study will be useful for finding the most efficient sgRNAs for restoring dystrophin disrupted by a specific mutation. Whereas NGS is also able to find the best sgRNAs, it is time consuming and expensive, especially when the number of the candidate sgRNAs is large. The GFP-reporter cells can not only be used to find the most efficient sgRNAs, but also be used for convenient and quantitative Cas9 editing assay during therapy development.

## Materials and methods

### Constructs

Constructs for expressing Cas9 and various guide RNAs are described in S1 Table. Plasmids used for packaging RNPs into lentiviral capsids were described in our previous papers [27, 40]. Plasmids are available up on request. Target sequences and oligos for making sgRNA expression plasmids are listed in S2 Table. Gene synthesis was done by GenScript Inc. All constructs made in this laboratory were verified by DNA sequencing.

#### Animals

The hDMDdel52/*mdx* mice have been described recently and are kind gifts from Annemieke Aartsma-Rus and Maaike van Putten [22]. After arrival the mice were housed in the pathogen-free animal facility at Wake Forest University Health Sciences. Experiments were conducted in accordance with the National Research Council publication Guide for Care and Use of Laboratory Animals, and approved by the Institutional Animal Care and Use Committee of Wake Forest University Health Sciences (Animal protocol number A18-087). Mice were kept in microisolator cages with 12-h light/dark cycles and were fed *ad libitum*. A total of twenty mice were used in the study. Carbon dioxide (CO2) overdose, which causes rapid unconsciousness followed by death, was used to euthanize mice. This method of euthanasia is a rapid, painless, stress-free death and is the most common mice euthanasia method. The mice were exposed to CO2 without being removed from their home cage, so that the animals were not stressed by handling or being moved to a new environment. The CO₂ flow rate was set to displace 10% to 30% of the cage volume per minute. When the mice showed deep narcosis, they were subjected to cervical dislocation as a secondary method of euthanasia. After euthanasia, muscle tissues were collected for muscle progenitor cell isolation.

#### Generating GFP-reporter cells for sgRNA screening

HEK293T cells (ATCC^®^ CRL-3216^™^, used between 30–36 passages) were cultured in DMEM with 10% FBS, and 100 U/ml penicillin and 100 μg/ml streptomycin (ThermoFisher Scientific). Mycoplasma was checked with the MycoAlert^™^ Mycoplasma Detection Kit (Lonza). HEK293T cells were used to make lentiviral vectors expressing a *DMD* exon 53 GFP-reporter cassette (S1 Table for information about lentiviral vector pSin-EF2-DMD53-GFP). The GFP expression from the reporter cassette is disrupted by insertion of a 74 nt after the start codon of GFP to disrupt the reading frame. GFP will be expressed if gene editing in the inserted sequence restores the GFP reading frame. To make the GFP-reporter lentiviral vector, 5x10^6^ HEK293T cells were seeded in 10-cm dishes in DMEM growth medium 24 hours before transfection. On the day of transfection, 9 μg pSin-EF2-DMD53-GFP plasmid DNA was mixed with 6 μg psPAX2 and 3 μg pMD2G DNA in 0.5 ml OPTI-MEM. In another tube, 54 μl Fugene 6 was added into 0.5 ml OPTI-MEM. The DNA mixture and the Fugene 6 mixture were combined and incubated at room temperature for 15 mins before they were added into the cells. Twenty four hours after transfection, the medium was changed to DMEM with 10% FBS, and the supernatant was collected 24 hours after medium change. The supernatant containing lentiviral vectors was diluted by equal volume of DMEM medium and used to transduce rapidly dividing HEK293T cells in the presence of 6 μg/ml polybrene. 48 hours after transduction, the cells were incubated in DMEM medium containing 10% FBS and 2 μg/ml puromycin for 7 days to kill the cells without vector DNA integration. The puromycin-resistant cells were used in GFP-reporter assays. The DMD53 GFP-reporter cells were authenticated once every 6 months by sequencing the GFP-reporter cassette region.

#### Packaging of Cas9 ribonucleoprotein (RNP) in Lentivirus-like particles (LVLP)

Packaging of Cas9 RNPs in LVLPs was achieved by three plasmids co-transfection of HEK293T cells as we have recently described [29]. The three plasmids include the aptamer binding protein modified packaging plasmid psPAX2-D64V-NC-COM, envelope plasmid pMD2.G (Addgene #12259), and the target plasmid co-expressing Cas9 and various sgRNA (S1 Table). To produce LVLPs packaged with RNPs, HEK293T cells were transfected with mixed DNA of the three plasmids using the procedure described above. 24h after transfection, the medium was changed to Opti-MEM and the LVLPs were collected twice with one collection each 24 h. The supernatant was spun for 10 min at 500 g to remove cell debris. The supernatants were used directly or were concentrated as described below.

#### Concentrating LVLPs

The supernatants were concentrated with the KR2i TFF System (KrosFlo^®^ Research 2i Tangential Flow Filtration System) (Spectrum Lab, Cat. No. SYR2-U20) using the concentration-diafiltration-concentration mode. Typically, 150–300 ml supernatant was first concentrated to about 50 ml, diafiltrated with 500 ml to 1000 ml PBS, and finally concentrated to about 8 ml. The hollow fiber filter modules were made from modified polyethersulfone, with a molecular weight cut-off of 500 kDa. The flow rate and the pressure limit were 80 ml/min and 8 psi for filter module D02-E500-05-N, and 10 ml/min and 5 psi for the filter module C02-E500-05-N. Since the TFF method produced lentivirus and LVLPs with the best activities, data were generated with virus or LVLPs concentrated by the TFF system unless otherwise stated.

#### LVLP quantification

Particle concentrations were determined by p24 (lentiviral capsid protein CA) based ELISA (Cell Biolabs, QuickTiter^™^ Lentivirus Titer Kit Catalog Number VPK-107). When unpurified samples were assayed, the viral particles were precipitated according to the manufacturer’s instructions so that the soluble p24 peptide was not detected.

#### Lentiviral vector and LVLP transduction

Lentiviral vector or LVLPs (ng p24 protein) were added to 2.5x10^4^ cells grown in 24-well plates, with 8 μg/ml polybrene. The cells were incubated with the particle-containing medium for 12–24 hours, after which normal medium was replaced.

#### Flow cytometry analysis of GFP-positive cells after gene editing-mediated GFP reading frame restoration

The *DMD* exon 53 GFP reporter cells (HEK293T derived) described above were used to detect the reframing activity of sgRNAs targeting *DMD* exon 53. The GFP-reporter cells expressed no EGFP due to the disruption of the EGFP reading frame by inserting *DMD* exon 53 target sequences after the GFP start codon. INDELs formed after gene editing may restore the EGFP reading frame, resulting in EGFP expression. GFP-positive cells were analyzed by fluorescence microscopy or flow cytometry (BD Biosciences, Accuri C6) 48 hours after DNA transfection or LVLP transduction [40].

#### Isolating and culturing mouse myoblasts

Muscle progenitor cells were isolated from a 4-week old male mouse as described previously [41, 42]. The hDMDdel52/*mdx* mice contain a mutated mouse dystrophin gene and a human *DMD* gene with exon 52 deleted [22]. Muscle tissues from the hind limbs, including the tibialis anterior (TA), soleus, gastrocnemius, quadriceps, and triceps muscles were collected and small pieces were digested in 0.2% collagenase I (Worthington #4196, 200-300U/mg) in serum free DMEM (0.1–0.15g tissue per ml) for 1 hour at 37 degrees Celcius. After neutralization by adding twice the original volume of DMEM (low glucose) with 10% FBS, the tissues were spun down and washed once with proliferation medium [DMEM with 20% FBS, 10% horse serum, 1% chicken embryo extract (Sera laboratory), 1% Antibiotic/Antimycotic solution (HyClone) and 5ng/ml bFGF). The digested tissue pellet was re-suspended and cultured in Matrigel (BD 354234, stock solution 10mg/ml) coated 6-well plate containing 4ml/well proliferation medium. For coating, dilute 1 volume of Matrigel stock solution with 5 volume of PBS and add 0.5 ml diluted solution to each well and coat for at least 2 hours at 37 degree. After 3–5 days the progenitor cells grew out of the fibers. The attached cells were collected at 50–60% confluence and frozen for future use.

#### Gene editing in myoblasts

Myoblasts isolated as described above were seeded in Matrigel coated 12-well tissue culture plate (coat with 1:200 diluted Matrigel stock solution at 30 degree for 2 hours) at a density of 10^5^ cells/well. To transduce myoblasts with lentivirus-like particles, 40–400 ng p24 of particles were mixed with 2.5 ml of muscle progenitor proliferation medium and polybrene (final concentration 8 μg/ml) and the mixture was added to the progenitor cells. Twelve to sixteen hours later the particles were removed by changing to fresh proliferation medium. For myotube differentiation, the cells were cultured in differentiation medium [DMEM with 2% Horse serum and 1% Antibiotic/Antimycotic solution (HyClone)] for about one week. The cells were collected for genomic DNA or RNA extraction to detect gene editing on the target site.

#### RNA isolation and reverse transcription

A RNeasy Plus Mini Kit (QIAGEN) was used to isolate RNA from cells. The QuantiTect Reverse Transcription Kit (QIAGEN) was used to reverse-transcribe the RNA to cDNA following the instructions of the kits.

#### Next-generation sequencing and data analyses

Genomic DNA was isolated from DMD53 GFP-reporter cells, HEK293T cells or myoblasts (isolated from mice as described above) with the DNeasy Blood & Tissue Kit (Qiagen). Three different DNA regions were amplified for next-generation sequencing. The *DMD* exon 53 target sequence in the GFP-reporter cassette of GFP-reporter cells was amplified with primers reporter-mut-F1 and reporter-R2. The endogenous human *DMD* exon 53 target region was amplified with primers DMD53-F and DMD53-R. The cDNA sequence of human *DMD* exon 53 was amplified from cDNA with primers DMD51-EX-F and DMD54-R1, which could detect exon 53 skipping. The 100~350 bp PCR products were recovered from the gel (to exclude mouse *Dmd* cDNA derived amplicons (S2 Table for primer sequences).

Genomic DNA template input for PCR was up to 0.5 μg if possible. For samples with low DNA concentration, 0.2 μg of DNA was used. Predetermined minimal cycling numbers (25–30) were used to reduce amplification bias. The proofreading HotStart^®^ ReadyMix from KAPA Biosystems (Wilmington, MA) was used for PCR. The PCR products were purified with the NucleoSpin Gel and PCR Clean-Up kit (Takara) and NGS was done at Genewiz with the Amplicon-EZ service (GENEWIZ, Morrisville, NC). Sequence analyses were done with online software Cas-Analyzer [43] and crispresso2 [44] for mutation analyses.

## Statistical analysis

GraphPad Prism software (version 5.0, GraphPad Software Inc) was used for T-tests, chi-square analysis and Analysis of Variance (ANOVA). Tukey post hoc tests were performed following ANOVA to analyze data from more than two groups, and Bonferroni post hoc tests were performed following ANOVA in cases of two factors. *P*<0.05 was regarded as statistically significant.

## Supporting information

S1 Fig GFP-positive cells were generated after genome editing in the GFP-reporter cassette of the GFP-reporter cells. (DOCX)

S2 Fig Flow cytometry analysis of GFP-positive percentage. (DOCX)

S3 Fig NGS analysis of Sa-gRNA2 RNP generated INDELs in GFP-reporter cells. (DOCX)

S4 Fig NGS analysis of Sp-gRNA1 RNP generated INDELs in GFP-reporter cells. (DOCX)

S5 Fig NGS analysis of Sp-gRNA2 RNP generated INDELs in GFP-reporter cells. (DOCX)

S6 Fig Sequencing confirming the deletion of the DNA between the two target sites of Sa-50-g2 and Sa-51-g2. (DOCX)

S7 Fig hDMDdel52/*mdx* mice have background dystrophin expression due to spontaneous exon 53 skipping, which restores dystrophin reading frame. (DOCX)

S1 Table Constructs made for the study. (DOCX)

S2 Table Guide sequences and primers. (DOCX)
