# Supplementary material for: Sensitive and reliable evaluation of single-cut sgRNAs to restore dystrophin by a GFP-reporter assay
Source: PLoS One. 2020 Sep 24;15(9):e0239468. doi: 10.1371/journal.pone.0239468 (PMC7514106; doi:10.1371/journal.pone.0239468)
Supplement: S1 Fig — (DOCX) [file pone.0239468.s001.docx]

**
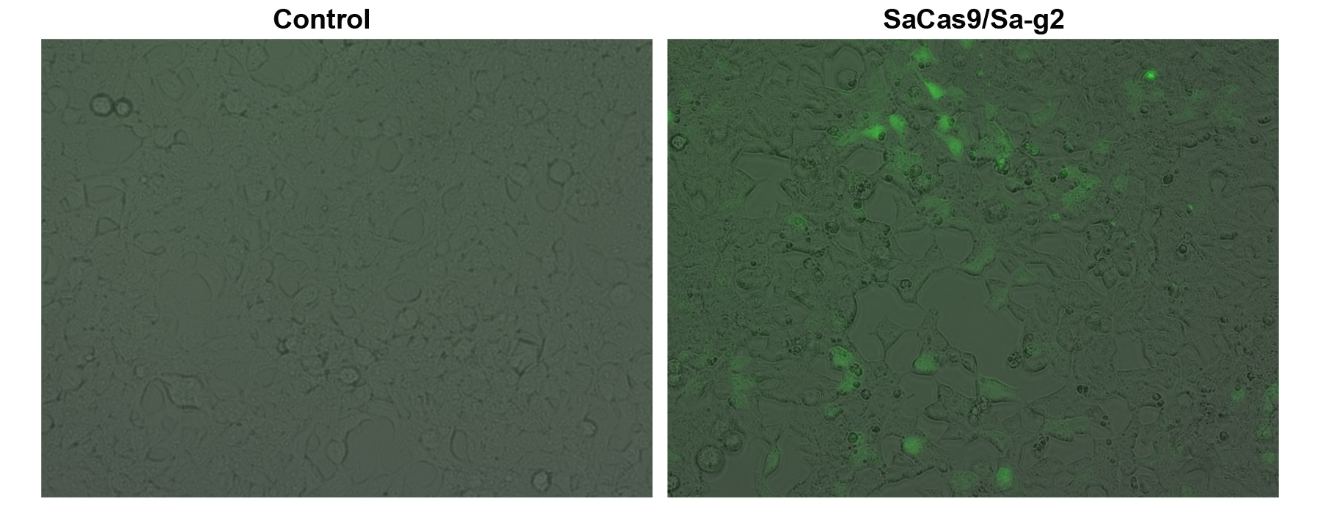
**

**S1 Fig**. GFP-positive cells were generated after genome editing in the GFP-reporter cassette of the GFP-reporter cells. Control cells were transfected with 500 ng of plasmid DNA expressing SaCas9 and sgRNA targeting *IL2RG*. GFP-positive cells were observed in cells transfected with DNA expressing 500 ng plasmid DNA expressing SaCas9 and sgRNA Sa-gRNA2.
