# Supplementary material for: Sensitive and reliable evaluation of single-cut sgRNAs to restore dystrophin by a GFP-reporter assay
Source: PLoS One. 2020 Sep 24;15(9):e0239468. doi: 10.1371/journal.pone.0239468 (PMC7514106; doi:10.1371/journal.pone.0239468)
Supplement: S2 Fig — (DOCX) [file pone.0239468.s002.docx]

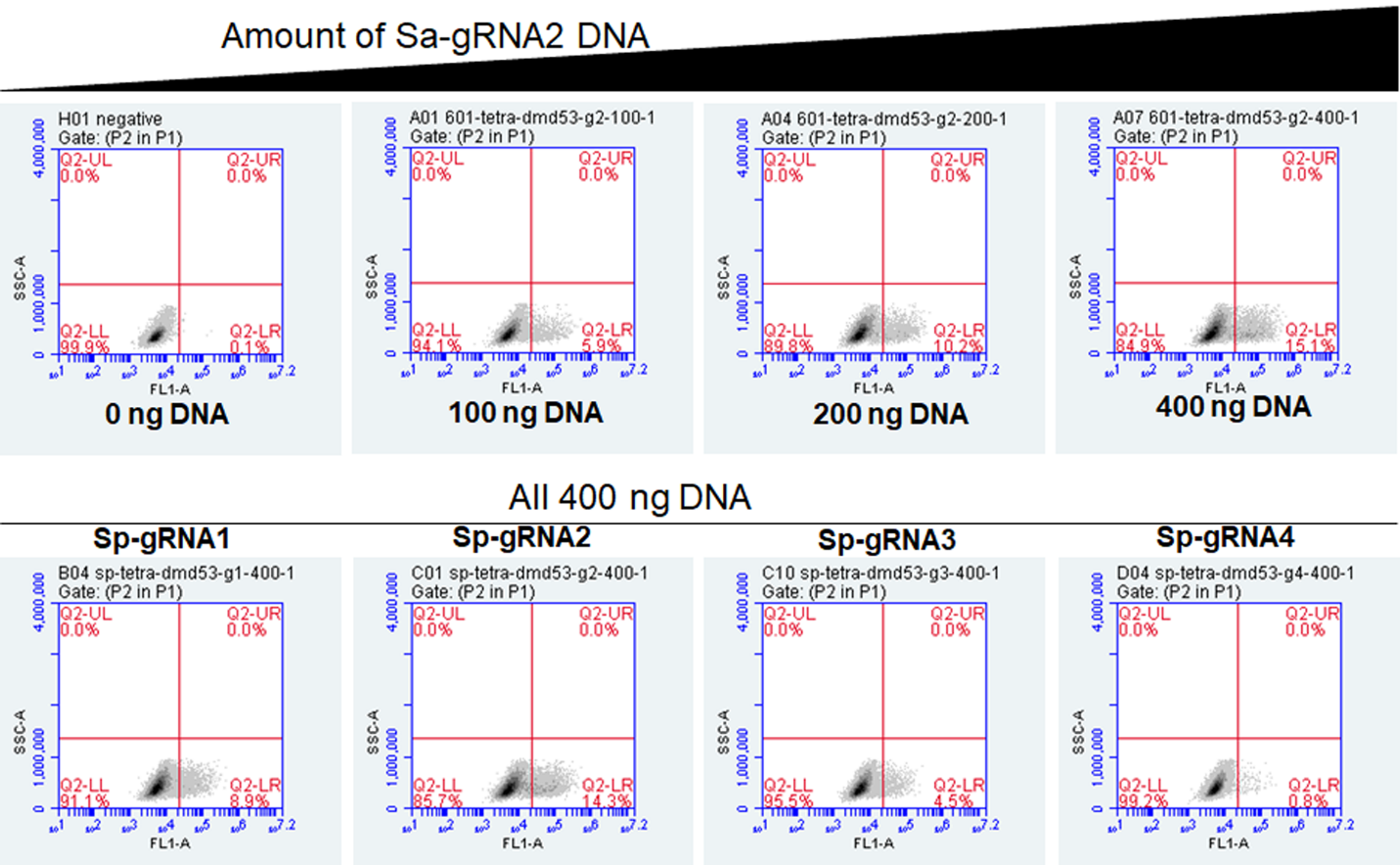


**S2 Fig**. Flow cytometry analysis of GFP-positive percentage. Top row: Increasing SaCas9/Sa-gRNA2 expressing DNA caused increased percentage of GFP-positive cells in the GFP-reporter cells. Bottom row: Same amount of DNA expressing different sgRNA generated different percentages of GFP-positive cells.
