# Supplementary figures and images for: Sensitive and reliable evaluation of single-cut sgRNAs to restore dystrophin by a GFP-reporter assay
Source: PLoS One. 2020 Sep 24;15(9):e0239468. doi: 10.1371/journal.pone.0239468 (PMC7514106; doi:10.1371/journal.pone.0239468)

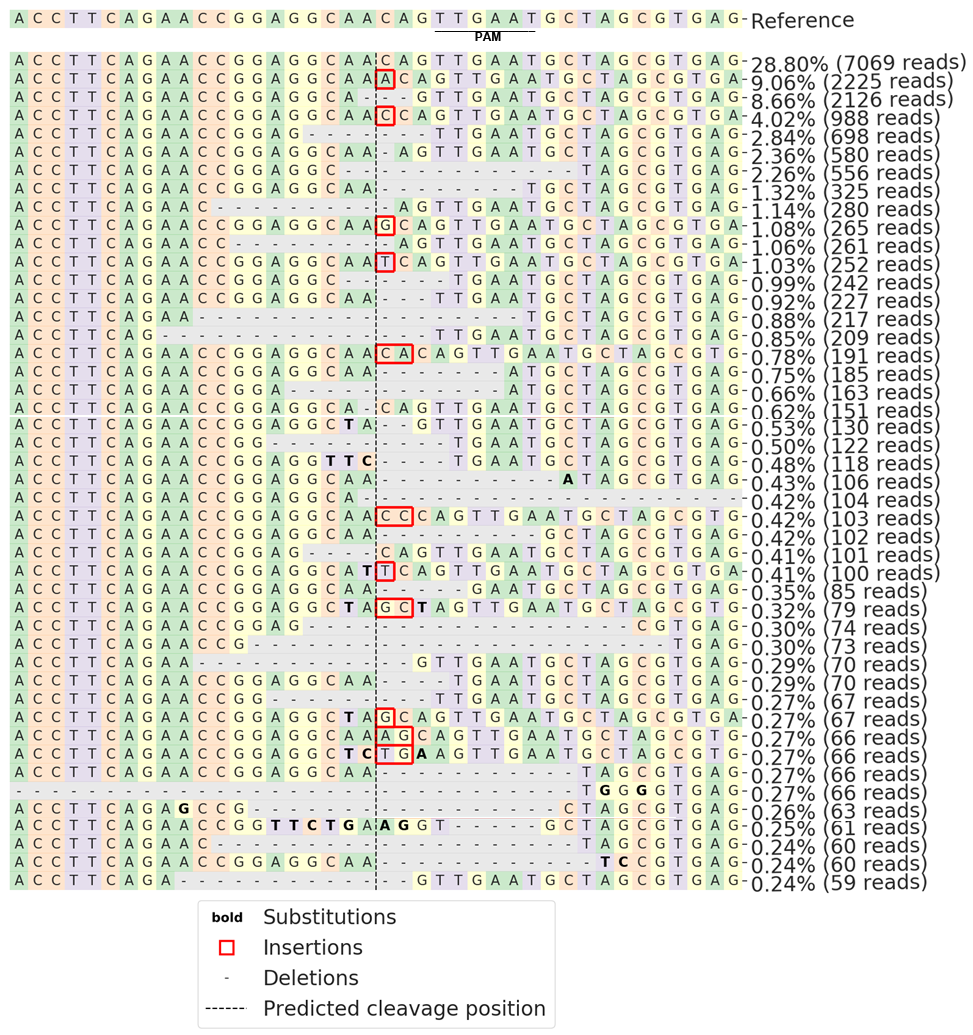


**S3 Fig**. NGS analysis of Sa-gRNA2 RNP generated INDELs in GFP-reporter cells.

Supplement: S3 Fig — (DOCX) [file pone.0239468.s003.docx]

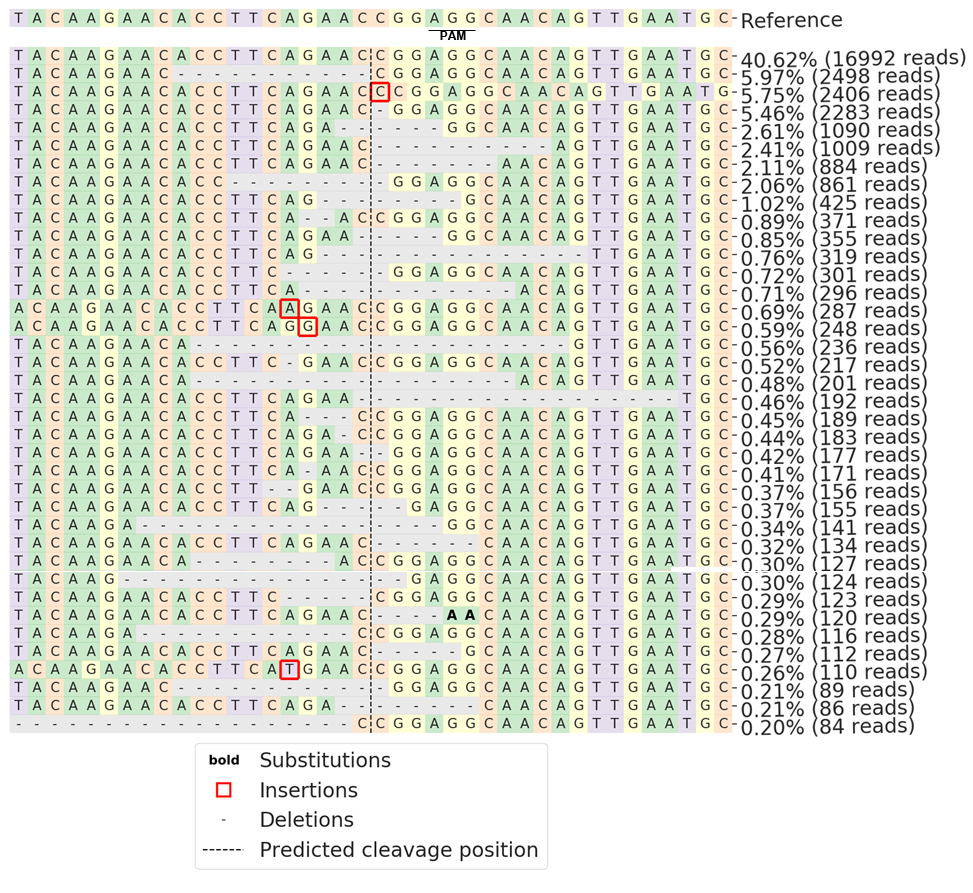


**S4 Fig**. NGS analysis of Sp-gRNA1 RNP generated INDELs in GFP-reporter cells.

Supplement: S4 Fig — (DOCX) [file pone.0239468.s004.docx]

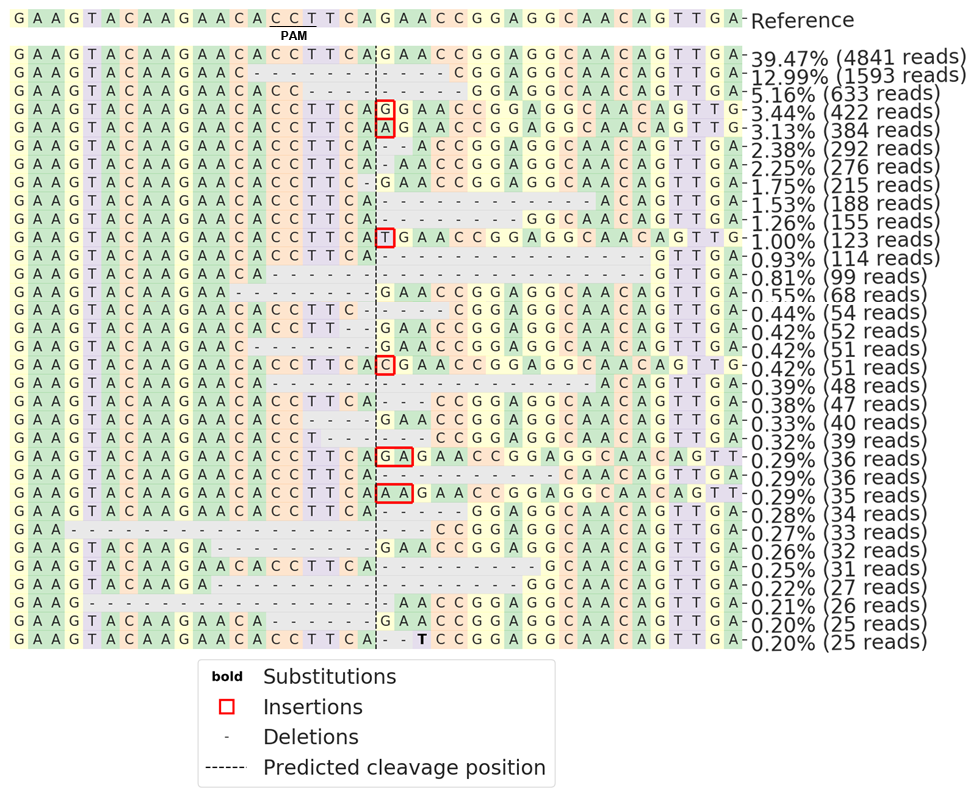


**S5 Fig**. NGS analysis of Sp-gRNA2 RNP generated INDELs in GFP-reporter cells.

Supplement: S5 Fig — (DOCX) [file pone.0239468.s005.docx]
