# Supplementary material for: Sensitive and reliable evaluation of single-cut sgRNAs to restore dystrophin by a GFP-reporter assay
Source: PLoS One. 2020 Sep 24;15(9):e0239468. doi: 10.1371/journal.pone.0239468 (PMC7514106; doi:10.1371/journal.pone.0239468)
Supplement: S6 Fig — (DOCX) [file pone.0239468.s006.docx]

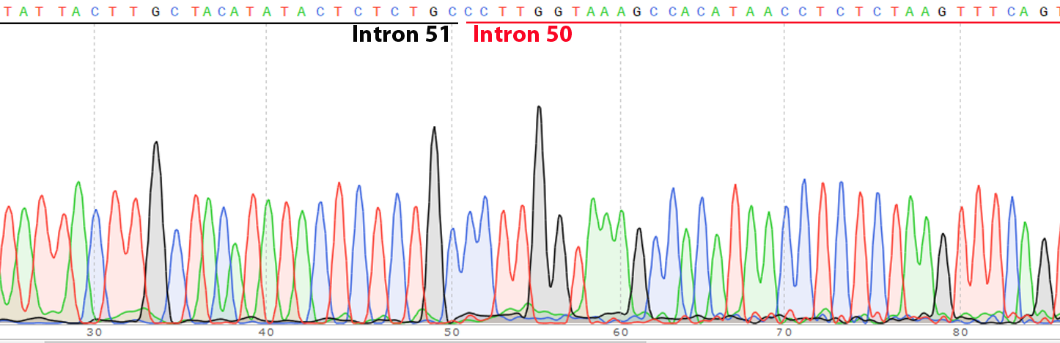


**S6 Fig**. Sequencing confirming the deletion of the DNA between the two target sites of Sa-50-g2 and Sa-51-g2. Primer DMD51-R2 was used for the sequencing. After deletion of the DNA between the two target sites, the sequence is: GCTGCTCTTTCTGGCATTGTCATACGTGTATTGCTTGTACTACTCACTGAATCTACACAACTGCCCTTATGACATTTACCCTGTTATTATTCCTCTTTTAAGGTAAATACATGAAAAATGCTTCCCACTTTGCCTTGCTTACTGCTTATTGCTAGTACTGAACAAATGTTAGAACTGAAACTTAGAGAGGTTATGTGGCTTTACCAAGGGCAGAGAGTATACGTAGCAAGTAATATATGTACTGCAAGCAATACATACTATTGCTGCGGTAATAACTGTAACTG.
