# Supplementary material for: Sensitive and reliable evaluation of single-cut sgRNAs to restore dystrophin by a GFP-reporter assay
Source: PLoS One. 2020 Sep 24;15(9):e0239468. doi: 10.1371/journal.pone.0239468 (PMC7514106; doi:10.1371/journal.pone.0239468)
Supplement: S7 Fig — (DOCX) [file pone.0239468.s007.docx]

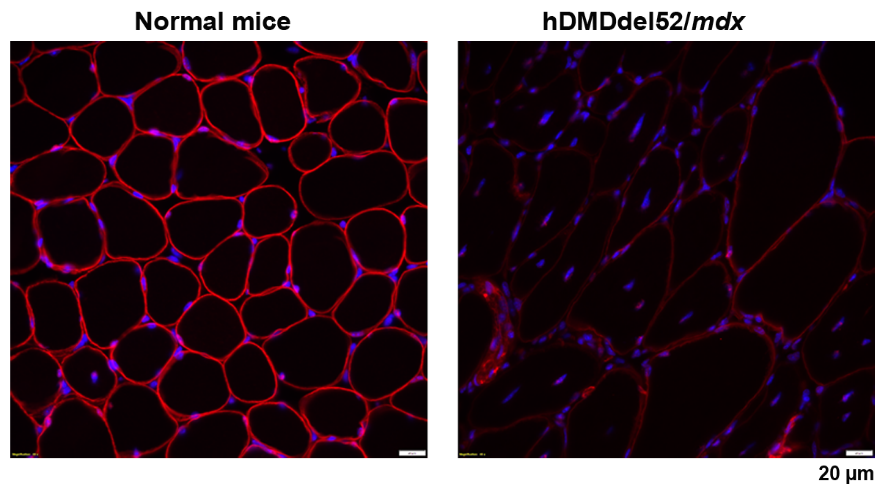


**S7 Fig**. hDMDdel52/*mdx* mice have background dystrophin expression due to spontaneous exon 53 skipping, which restores dystrophin reading frame. Mice were 1 month old. Red color shows dystrophin staining (Ab15277, 1:100), blue color shows the nuclei. In hDMDdel52/*mdx* mice there are nuclei in the middle of the muscle fibers, indicating muscle regeneration after damage.
