# Supplementary material for: Sensitive and reliable evaluation of single-cut sgRNAs to restore dystrophin by a GFP-reporter assay
Source: PLoS One. 2020 Sep 24;15(9):e0239468. doi: 10.1371/journal.pone.0239468 (PMC7514106; doi:10.1371/journal.pone.0239468)
Supplement: S1 Table — (DOCX) [file pone.0239468.s008.docx]

**S1 Table. Constructs made for the study**

| No. | Name | Purpose | Generation strategy |
| --- | --- | --- | --- |
| 1 | pSin-EF2-DMD 53-GFP | Lentiviral vector for making GFP-reporter cells. The cassette has 74 nt, of which 68 nt are from DMD exon 53, inserted after the start codon of GFP cDNA. | Two synthetic oligos 53-GFP-F (AATTCTGAgGCCACCATGTTGAAAGAATTCAGAATCAGTGGGATGAAGTACAAGAACACCTTCAGAACCGGAGGCAACAGTTGAATg) and 53-GFP-R (ctagcATTCAACTGTTGCCTCCGGTTCTGAAGGTGTTCTTGTACTTCATCCCACTGATTCTGAATTCTTTCAACATGGTGGCcTCAG) were annealed and ligated into the EcoRI and NheI sites of pSin-EF2-IL2RG-HBB-GFP ^1^. |
| 2 | pspCas9-3'UTR-Tetra-com-vector | Vector plasmid for expressing spCas9 and sgRNA. There are two copies of HBB 3’ UTR in the 3’UTR of spCas9 to enhance expression. The Tetraloop of the sgRNA scaffold was replaced by com-aptamer to enable Cas9 RNP encapsulation into viral capsids via com-COM interaction. | A synthesized U6-sgRNA expression cassette (Genscript) was inserted into the AflIII and Acc65I sites of pSpCas9-1loop-3’UTR ^2^. The sequence of the AflIII-Acc65I fragment was: ACATGTgagggcctatttcccatgattccttcatatttgcatatacgatacaaggctgttagagagataattggaattaatttgactgtaaacacaaagatattagtacaaaatacgtgacgtagaaagtaataatttcttgggtagtttgcagttttaaaattatgttttaaaatggactatcatatgcttaccgtaacttgaaagtatttcgatttcttggctttatatatcttgtggaaaggacgaaacaccggtgtcttcCTCGAGgaagacccGTTTGAGAGCTAggccCTGAATGCCTGCGAGCATCCCACggccTAGCAAGTTCAAATAAGGCTAGTCCGTTATCAACTTGAAAAAGTGGCACCGAGTCGGTGCTTTTTTTGGTACC. The resulted plasmid was cut with XbaI and re-ligated to remove the MS2 aptamer after spCas9 stop codon. |
| 3 | pspCas9-3’UTR-tetra-com-53-sp-g1 | Plasmid expressing SpCas9 and Sp-g1 sgRNA, with a com aptamer replacing the Tetra loop of the sgRNA scaffold. | The annealed products of 53-sp-g1-F (ACCGaagaacaccttcagaaccgg) and 53-sp-g1-R (AAACccggttctgaaggtgttctt) were inserted between the BbsI sites of pspCas9-3'UTR-Tetra-com-vector. |
| 4 | pspCas9-3’UTR-tetra-com-53-sp-g2 | Plasmid expressing SpCas9 and Sp-g2 sgRNA, with a com aptamer replacing the Tetra loop of the sgRNA scaffold. | The annealed products of 53-sp-g2-F (ACCGactgttgcctccggttctga) and 53-sp-g2-R (AAACtcagaaccggaggcaacagt) were inserted between the BbsI sites of pspCas9-3'UTR-Tetra-com-vector. |
| 5 | pspCas9-3’UTR-tetra-com-53-sp-g3 | Plasmid expressing SpCas9 and Sp-g3 sgRNA, with a com aptamer replacing the Tetra loop of the sgRNA scaffold. | The annealed products of 53-sp-g3-F (ACCGtacaagaacaccttcagaac) and 53-sp-g3-R (AAACgttctgaaggtgttcttgta) were inserted between the BbsI sites of pspCas9-3'UTR-Tetra-com-vector. |
| 6 | pspCas9-3’UTR-tetra-com-53-sp-g4 | Plasmid expressing SpCas9 and Sp-g4 sgRNA, with a com aptamer replacing the Tetra loop of the sgRNA scaffold. | The annealed products of 53-sp-g4-F (ACCGtttcattcaactgttgcctc) and 53-sp-g4-R (AAACgaggcaacagttgaatgaaa) were inserted between the BbsI sites of pspCas9-3'UTR-Tetra-com-vector. |
| 7 | pX601-Tetra-com-53-Sa-g1 | Plasmid expressing SaCas9 and Sa-g1 sgRNA, with a com aptamer replacing the Tetra loop of the sgRNA scaffold. | The annealed products of 53-g1-F (CACCgTTGAAAGAATTCAGAATCAG) and 53-g1-R (AAACCTGATTCTGAATTCTTTCAAc) were inserted between the BsaI sites of pX601-Tetra-com-vector. |
| 8 | pX601-Tetra-com-53-Sa-g2 | Plasmid expressing SaCas9 and Sa-g2 sgRNA, with a com aptamer replacing the Tetra loop of the sgRNA scaffold. | The annealed products of 53-g2-F (CACCGCTTCAGAACCGGAGGCAACAG) and 53-g2-R (AAACCTGTTGCCTCCGGTTCTGAAGC) were inserted between the BsaI sites of pX601-Tetra-com-vector. |
| 9 | pX601-Tetra-com-53-Sa-g3 | Plasmid expressing SaCas9 and Sa-g3 sgRNA, with a com aptamer replacing the Tetra loop of the sgRNA scaffold. | The annealed products of 53-g3-F (CACCGTTGTACTTCATCCCACTGATT) and 53-g3-R (AAACAATCAGTGGGATGAAGTACAAC) were inserted between the BsaI sites of pX601-Tetra-com-vector. |
| 10 | pX601-Tetra-com-50-2 | Plasmid expressing SaCas9 and 50-2 sgRNA (targeting human DMD intron 50), with a com aptamer replacing the Tetra loop of the sgRNA scaffold. | The annealed products of DMD-50-2-F (CACCgTATGTGGCTTTACCAAGGTCC) DMD-50-2-R (AAACGGACCTTGGTAAAGCCACATAc) were inserted between the BsaI sites of pX601-Tetra-com-vector. |
| 11 | pX601-Tetra-com-51-2 | Plasmid expressing SaCas9 and 51-2 sgRNA (sgRNA 84 (targeting human DMD intron 51), with a com aptamer replacing the Tetra loop of the sgRNA scaffold. | The annealed products of DMD-51-2-F (CACCGTGTTATTACTTGCTACTGCA) and DMD-51-2-R (AAACTGCAGTAGCAAGTAATAACAC) were inserted between the BsaI sites of pX601-Tetra-com-vector. |
| 12 | pspCas9-3’UTR-ST2-com-53-sp-g2 | Plasmid expressing SpCas9 and Sp-g2 sgRNA, with a com aptamer replacing the ST2 loop of the sgRNA scaffold. | The annealed products of 53-sp-g2-F (ACCGactgttgcctccggttctga) and 53-sp-g2-R (AAACtcagaaccggaggcaacagt) were inserted between the BbsI sites of pspCas9-3'UTR-ST2-com-vector. |
| 13 | pspCas9-3’UTR-ST2-com-1617 | Plasmid expressing SpCas9 and a sgRNA targeting human *BCL11A* enhancer sequence (sgRNA-1617), with a com aptamer replacing the ST2 loop of the sgRNA scaffold. | The annealed products of globin1617-F (accgGCTAACAGTTGCTTTTATCAC  ) and globin1617-R (aaacGTGATAAAAGCAACTGTTAGC) were inserted between the BbsI sites of pspCas9-3'UTR-ST2-com-vector. |

1. Javidi-Parsijani, P, Niu, G, Davis, M, Lu, P, Atala, A, and Lu, B (2017). No evidence of genome editing activity from Natronobacterium gregoryi Argonaute (NgAgo) in human cells. *PLoS One* **12**: e0177444.

2. Lu, B, Javidi-Parsijani, P, Makani, V, Mehraein-Ghomi, F, Sarhan, WM, Sun, D*, et al.* (2019). Delivering SaCas9 mRNA by lentivirus-like bionanoparticles for transient expression and efficient genome editing. *Nucleic Acids Res* **47**: e44.
