# Supplementary material for: Sensitive and reliable evaluation of single-cut sgRNAs to restore dystrophin by a GFP-reporter assay
Source: PLoS One. 2020 Sep 24;15(9):e0239468. doi: 10.1371/journal.pone.0239468 (PMC7514106; doi:10.1371/journal.pone.0239468)
Supplement: S2 Table — (DOCX) [file pone.0239468.s009.docx]

**S2 Table. Guide sequences and primers**

| **Name** | **Guide sequence** | **PAM** | **Forward oligo** | | **Reverse oligo** |
| --- | --- | --- | --- | --- | --- |
| **For single-cut dystrophin restoring via targeting exon 53** | | | | | |
| Sa-gRNA1 | TTGAAAGAATTCAGAATCAG | TGGGAT | CACCgTTGAAAGAATTCAGAATCAG | | AAACCTGATTCTGAATTCTTTCAAc |
| Sa-gRNA2 | CTTCAGAACCGGAGGCAACAG | TTGAAT | CACCGCTTCAGAACCGGAGGCAACAG | | **AAACCTGTTGCCTCCGGTTCTGAAGC** |
| Sa-gRNA3 | TTGTACTTCATCCCACTGATT | ATTCAG | CACCGTTGTACTTCATCCCACTGATT | | AAACAATCAGTGGGATGAAGTACAAC |
| Sp-gRNA1 | AAGAACACCTTCAGAACCGG | AGG | ACCGaagaacaccttcagaaccgg | | AAACccggttctgaaggtgttctt |
| Sp-gRNA2 | ACTGTTGCCTCCGGTTCTGA | AGG | ACCGactgttgcctccggttctga | | AAAC**tcagaaccggaggcaacagt** |
| Sp-gRNA3 | TACAAGAACACCTTCAGAAC | CGG | ACCGtacaagaacaccttcagaac | | AAACgttctgaaggtgttcttgta |
| Sp-gRNA4 | tttcattcaactgttgcctc | CGG | ACCGtttcattcaactgttgcctc | | AAACgaggcaacagttgaatgaaa |
| **For targeting introns 50 and 51 to remove exon 51** | | | | | |
| 50-2 gRNA | TATGTGGCTTTACCAAGGTCC | CAGAGT | CACCgTATGTGGCTTTACCAAGGTCC | | AAACGGACCTTGGTAAAGCCACATAc |
| 51-2 gRNA | GTGTTATTACTTGCTACTGCA | GAGAGT | CACCGTGTTATTACTTGCTACTGCA | | AAACTGCAGTAGCAAGTAATAACAC |
| Globin 1617-gRNA | GCTAACAGTTGCTTTTATCAC | AGG | accgGCTAACAGTTGCTTTTATCAC | | aaacGTGATAAAAGCAACTGTTAGC |
| **Primers** | | | | | |
| **Name** | | **Sequence** | | **Usage** | |
| Reporter-mut-F1 | | tccatttcaggtgtcgtgag | | Amplifying the target sequence in the reporter cassette for NGS | |
| Reporter-R2 | | GAACTTCAGGGTCAGCTTGC | |  |  |
| DMD53-F | | TCCTGTTGTTCATCATCCTAGC | | Amplifying the endogenous targets in *hDMD* exon 53 for NGS | |
| DMD53-R | | TCCAGCCATTGTGTTGAATC | |  |  |
| DMD51-EX-F | | ggcttggacagaacttaccg | | Amplifying the *hDMD* cDNA from mouse myoblasts for NGS | |
| DMD54-R1 | | cactggcggaggtctttg | |  |  |
| DMD-50F | | GCTGCTCTTTCTGGCATTG | | For PCR detecting the DNA deletion caused by sgRNA 50-2 and 51-2 | |
| DMD-51R2 | | CAGTTACAGTTATTACCGCAGCA | |  |  |
